# Precision genome engineering in lactic acid bacteria

**DOI:** 10.1186/1475-2859-13-S1-S10

**Published:** 2014-08-29

**Authors:** Jan Peter van Pijkeren, Robert A Britton

**Affiliations:** 1 Department of Food Science, University of Wisconsin-Madison, Madison, Wisconsin 53706, USA; 2 Department of Microbiology and Molecular Genetics, Michigan State University, East Lansing, MI 48824, USA

## Abstract

Innovative new genome engineering technologies for manipulating chromosomes have appeared in the last decade. One of these technologies, recombination mediated genetic engineering (recombineering) allows for precision DNA engineering of chromosomes and plasmids in *Escherichia coli*. Single-stranded DNA recombineering (SSDR) allows for the generation of subtle mutations without the need for selection and without leaving behind any foreign DNA. In this review we discuss the application of SSDR technology in lactic acid bacteria, with an emphasis on key factors that were critical to move this technology from *E. coli *into *Lactobacillus reuteri *and *Lactococcus lactis*. We also provide a blueprint for how to proceed if one is attempting to establish SSDR technology in a lactic acid bacterium. The emergence of CRISPR-Cas technology in genome engineering and its potential application to enhancing SSDR in lactic acid bacteria is discussed. The ability to perform precision genome engineering in medically and industrially important lactic acid bacteria will allow for the genetic improvement of strains without compromising safety.

## Introduction

Genetics has been, and will continue to be, an essential tool for providing insight into molecular and biological function in all forms of life. For example, the temperature-sensitive lethal genetic screens performed in the 1960s and 70s in microbes were essential initial steps in providing a foundation for our current understanding of how DNA replication, transcription, and translation take place within the cell. Many of the molecular genetic tools in use today were originally developed in model organisms, such as *Escherichia coli *and *Bacillus subtilis*. Over the last 25 years many of these tools have been successfully adapted for use in lactic acid bacteria (LAB), including suicide plasmids for generating gene disruptions and the development of inducible expression systems for regulated protein production. For more information on tools currently in use in lactic acid bacteria, readers are directed to the following reference as a starting point [1]. In this review, we will highlight a newly developed technology, single-stranded DNA recombineering, which allows for precision genome engineering of bacteria. Recombineering and other emerging genome engineering tools can generate bacterial strains that are genetically indistinguishable from an organism whose genome has been altered by a natural selection procedure. A genetically modified organism (GMO) generated by precision genome engineering will blur the lines between the safety profiles of organisms traditionally considered as genetically modified organisms (GMO) and those organisms that have genetically altered genomes but are not considered GMO.

## Review

### Introduction to recombineering technology

In the past decade, the use of phage-encoded recombinases has led to multiple applications of recombineering (recombination-mediated genetic engineering) for *in vivo *genetic engineering in *Escherichia coli*. Two forms of recombineering, denoted here as double-stranded DNA (dsDNA) and single-stranded DNA (ssDNA) recombineering, have been described [2]. Initial studies focused on the use of the phage-derived lambda *red *locus, which consists of two genes involved in recombination (*exo, beta*), and subsequently two genes from the Rac prophage (*recE *and *recT*) (for review see [3]). Beta and RecT are ssDNA binding proteins that promote the annealing of complementary DNA and are often referred to as recombinases. Exo and RecE are exonucleases required to process dsDNA molecules to ssDNA intermediates for binding by Beta or RecT, respectively. dsDNA recombineering has been used for several different applications, including the insertion of DNA using as little as 50 bp of flanking homology on each end and subcloning DNA fragments by recombineering. This approach has been shown in *E. coli *to generate insertions up to 2.5 kb in length [4]. As dsDNA recombineering is not the subject of this review we direct readers to the following references (and references therein) for more information [2,5-7].

ssDNA recombineering (SSDR) allows for the engineering of subtle mutations in the chromosome without the need for any type of selection. The only requirements for SSDR are the inducible expression of a recombinase (Beta or RecT) and the ability to transform an oligonucleotide into the cell that harbors the desired base changes to be incorporated into the chromosome. When performed under appropriate conditions in *E. coli*, up to 50% of cells that are electroporated with the oligonucleotide will incorporate the mutation into the chromosome, obviating the need for antibiotic selection to recover the mutation [8-10]. Recombinants are easily identified using techniques such as MAMA-PCR, PCR coupled with restriction digest if the mutation alters a restriction site, or PCR amplification and sequencing after colony purification (see below).

To achieve such high efficiencies in *E. coli*, several key aspects of oligonucleotide design needed to be optimized. First, it was necessary to generate mutations that avoid the mismatch repair (MMR) system, which (in *E. coli*) can be achieved by having three or more consecutive base pairs altered or creating an oligonucleotide in which a C·C mismatch is generated [8,9]. Such mutations are poorly recognized by MMR and successfully avoiding MMR can increase SSDR by approximately 100-fold [8]. Second, for optimal levels of SSDR, oligonucleotides must be identical to the lagging strand of DNA synthesis, which is replicated as short Okazaki fragments in a discontinuous manner [11,12]. This is likely due to the fact that during synthesis of the lagging strand there is much more template DNA available for binding than found on the continuous leading strand synthesis template. Although it is not entirely clear how oligonucleotides are incorporated into the chromosome, it is possible that they either serve as primers for Okazaki fragment synthesis or they are ligated into the DNA as if being recognized as an Okazaki fragment. Third, modification of the oligonucleotide to avoid degradation of host exonucleases can improve SSDR efficiency. Placing phosphorothioate linkages at the 5' end of the oligonucleotide has been shown to improve SSDR in several studies [13-17].

Although SSDR has been an extremely successful tool for use in *E. coli*, there has been relatively little success in establishing recombineering at the necessary efficiencies to generate unselected mutations in other bacteria. However, the presence of *recT *homologs throughout bacteria, and the ability of Gram-positive RecT proteins to function as well as Beta in *E. coli *[18], suggested that it should be possible to establish SSDR in a wide range of organisms. Below, we describe the application of SSDR in two different lactic acid bacterial species, *Lactobacillus reuteri *and *Lactococcus lactis*. In both species, subtle mutations, yielding an in-frame stop codon or a single amino acid change, can be introduced into the chromosome at efficiencies ranging from 0.3-20%, depending on the chromosomal location. We will summarize the salient features of SSDR in *L. reuteri *and *L. lactis *and highlight key differences that need to be considered when attempting to establish SSDR in other LAB species. Several parameters needed to be optimized to establish efficient SSDR in *L. reuteri *ATCC PTA 6475 and *L. lactis *NZ9000, which we expect will serve as a blueprint for establishing this technology in other Gram-positive bacteria. Topics discussed below will include the minimal requirements to establish SSDR, followed by a detailed description of the three key parameters that need to be taken into consideration when designing oligonucleotides for SSDR in lactic acid bacteria in order to implement this technology in your organism of choice at levels that do not require selection strategies to identify a mutant genotype.

### SSDR in lactic acid bacteria

#### Choosing a suitable source of recombinase for SSDR

Phage-derived single-stranded DNA binding proteins are highly divergent in both their amino acid composition as well as their activity. Elegant work from Donald Court's group showed that recombinases from a phylogenetically diverse set of hosts displayed activity in *E. coli *[18]. Remarkably, a RecT protein isolated from the lactic acid bacterium *Enterococcus faecalis *yielded similar level of recombinants compared to phage Lambda-derived Beta protein in *E. coli*. This finding suggested that recombinases in LAB are as active as those previously characterized in *E. coli *and provided an impetus for screening for active recombinases in lactobacilli. *L. reuteri *6475 encodes two RecT proteins, and each has high-level activity in SSDR when produced *in trans *from an expression vector [19]. However, it is not necessarily best to use a native RecT when establishing SSDR in your organism of choice. We found it is not an absolute requirement to use the *L. reuteri *RecT proteins for SSDR in *L. reuteri *as we have demonstrated that the *E. faecalis *RecT protein has equal activity as the endogenous RecT proteins in *L. reuteri *6475. In addition, in *L. plantarum *BAA-793 expression of the endogenous *L. plantarum *RecT protein yielded many fewer recombinants compared to when RecT derived from *L. reuteri *6475 was expressed (unpublished data). Therefore, RecT isolated from different sources may need to be tested to identify the most active recombinase in a particular strain background.

#### Transformation efficiency

To introduce single-stranded DNA (*i.e*. oligonucleotides) into the cell, an efficient electroporation protocol must be available for the bacterium of interest. Although the efficiency at which ssDNA enters the cells may be different compared to plasmid DNA, it is likely that when plasmid DNA can cross the membrane, oligonucleotides can likely do the same. Therefore, establishment of an electroporation protocol may be done with plasmid DNA but at a later stage electroporation settings may be optimized with an oligonucleotide that, when incorporated in the chromosome, yields a selectable phenotype such as rifampicin-resistance (see below for additional details) to quantify the level of recombinants as a function of electroporation settings. Although we did not assess the minimum transformation efficiency required, in *L. lactis *a transformation efficiency of 10^5 ^colony forming units per microgram (cfu/µg) of plasmid DNA yielded sufficient levels of oligonucleotide in the cell to isolate mutations without the need for antibiotic selection. Because the transformation efficiency of *L. reuteri *6475 was poor when we started, we optimized the transformation efficiency from 10^3 ^to 10^6 ^cfu/µg plasmid DNA prior to attempting SSDR. Further improvement of transformation efficiency to 10^7 ^cfu/µg plasmid DNA did not result in a further increase in recombineering efficiency, suggesting that either the SSDR efficiency or the level of oligonucleotide that entered the cell had saturated.

#### Inducible expression of the recombinase

Once oligonucleotides enter the cell, optimal levels of the recombinase should be present to interact with the oligonucleotide to promote annealing with the template strand to form a complex for incorporation. We found in *L. reuteri *6475 that controlled expression of *recT *is key for efficient oligonucleotide incorporation [19]. In *E. coli *it was established that high levels of Beta expression for a prolonged period of time resulted in reduced cell viability, which we also found to be true for prolonged RecT expression in *L. reuteri *6475 [20]. A constitutive, moderate level of expression of RecT in *L. reuteri *6475 was well tolerated but did not yield high levels of recombinants. Only when RecT was induced for a short period of time during growth levels of SSDR were sufficient to isolate mutations without the need for selection. Another reason to titrate the levels of RecT is that in *E. coli *prolonged expression of Beta may increase unwanted secondary mutations [21]. Thus, identifying a system that allows controlled expression of RecT is required for optimal SSDR.

A few inducible expression systems, both relying on quorum sensing mechanisms to drive expression, have been described for use in LAB, including the well-established NICE (*ni*sin *c*ontrolled gene *e*xpression) system and a sakacin-based system from *Lactobacillus sakei *[22-24]. Although both systems were extremely useful in a variety of LAB strains to establish SSDR, not all strains are compatible with these expression systems. Therefore, the development of a general expression platform based on riboswitches, for example, may have merit for use in LAB to extend SSDR to other LAB strains.

#### Oligonucleotide design considerations

##### Lagging strand

Regardless of the target location in the bacterial chromosome, an oligonucleotide for SSDR is most efficiently incorporated when the oligonucleotide sequence is identical to the lagging strand of replication [11]. The lagging strand bias in *E. coli, Mycobacterium, L. reuteri*, and *L. lactis *is approximately 30-fold, 10,000-fold, 4,000-fold and 25-fold, respectively compared to mutations generated with a leading strand oligonucleotide [10,11,16,25]. Why *L. reuteri *and *Mycobacterium *are more sensitive to lagging strand bias than the other two strains is not clear. Thus it is important to be able to identify which strand is the lagging strand of DNA replication for optimal oligonucleotide design. Additional file 1 illustrates how one can identify the lagging strand of replication, and provides detail on oligonucleotide design to incorporate an in-frame stop codon in a target sequence.

##### Evasion of the mismatch repair system

Since a recombineering oligonucleotide that anneals to the template of the lagging strand at the replication fork would be considered part of the newly synthesized strand, the mismatch repair (MMR) machinery will preferentially repair the incoming oligonucleotide sequence back to wild-type. This was confirmed in *E. coli *by showing the use of mutants defective for MMR yielded large increases in SSDR efficiency [8,9]. However, it is undesirable to perform recombineering in MMR mutants because many unwanted mutations will occur at other sites in the genome. To circumvent this problem, oligonucleotides that generate mismatches that are not well recognized by MMR can be designed to mimic the increase in efficiency observed in MMR mutants [8]. In *E. coli*, the hierarchy for the efficiency of MMR to repair mismatches is reported as G·T, A·C, A·A G·G > T·T, T·C, A·G > C·C [8]. We observed that a similar hierarchy of MMR exists in *L. reuteri *6475 (van Pijkeren *et al*. manuscript in preparation); however, in *L. reuteri *6475 the efficacy of repair seems to be highly dependent on sequence context whereby the sequence upstream and downstream of the mismatch(es) may impact the efficacy of MMR. Although a single C·C mismatch may evade the MMR system in *L. reuteri *6475 (van Pijkeren *et al*. manuscript in preparation) it becomes problematic to identify a mutant when the mutation does not allow for selection (see section *Methods to screen for mutations when no selection is performed*). The most straightforward approach to evade mismatch repair in both *L. reuteri *and *L. lactis *is to design an oligonucleotide that results in 4 or 5 adjacent mismatches when annealed to the lagging strand template. We have shown in a MMR-deficient derivative of *L. reuteri *6475 that an oligonucleotide containing 5 adjacent mismatches yields similar levels of recombinants compared to the MMR deficient strain, suggesting that MMR does not recognize multiple consecutive mismatches in *L. reuteri*. Three adjacent mismatches can evade the MMR but only when cells are transformed with high concentrations of oligonucleotide (van Pijkeren *et al*. manuscript in preparation).

##### Oligonucleotide concentration

In addition to having an oligonucleotide that is identical to the lagging strand of replication and can evade mismatch repair, a third equally important parameter is the amount of oligonucleotide that is transformed into the cell. In *L. reuteri *6475 we found there is a strong linear and positive correlation between the amount of oligonucleotide transformed and the number of recombinants obtained when transforming recombineering oligonucleotide within the range of 1-100µg (37-3,780 pmol of oligonucleotide) [16,19]. Similarly for *L. lactis *NZ9000, we found that SSDR efficiency was linear, but this time up to 500µg of oligonucleotide was required for optimal SSDR activity. When optimizing the oligonucleotide concentration for SSDR in *L. reuteri *6475 and *L. lactis *NZ9000 we found that 200µg and 1 mg oligonucleotide causes lysis in the respective organisms, therefore we routinely use 100µg and 500µg oligonucleotide for SSDR in *L. reuteri *6475 and *L. lactis *NZ9000, respectively [16]. Importantly, the use of such high levels of oligonucleotide does not lead to increased mutation or insertion of the oligonucleotide at other sites in the genome [19]. We did not investigate the optimal oligonucleotide concentration in the other LAB tested but we were able to obtain recombinants when transforming 100µg oligonucleotide in *L. plantarum *BAA-793 and *L. gasseri *ATCC 33323, therefore this may be a good starting concentration when establishing SSDR in other LAB strains [19].

These oligonucleotide concentrations are in strong contrast to the oligonucleotide concentration used in *E. coli*, for example, in which recombination saturates with 5 pmol oligonucleotide [10], which represents approximately 10 oligonucleotide molecules per cell. Since approximately comparable levels of *L. reuteri *and *L. lactis *cells are transformed compared to *E. coli *(10^10 ^cfu in 100µl) we can conclude that *L. reuteri *6475 and *L. lactis *NZ9000 require approximately 750-fold and 3,750-fold more oligonucleotides per cell, respectively, to maximize SSDR efficiencies. We have not investigated what the rationale for this observation is but it is plausible that in LAB less oligonucleotide enters the cell due to the cell wall structure that is characteristic to Gram-positive bacteria.

#### Guide to establish SSDR in other LAB

**Figure 1:**
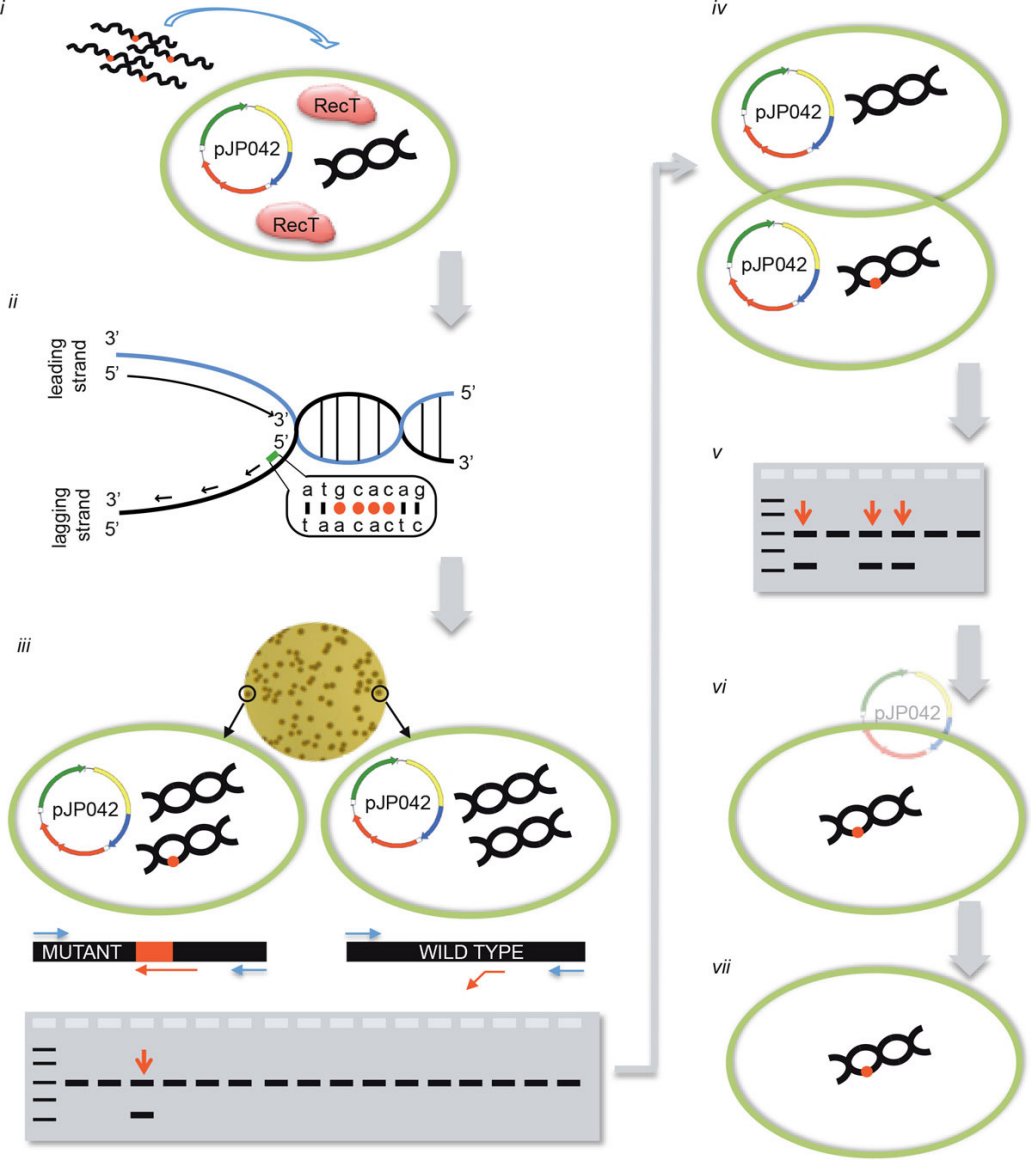
**Overview of recombineering in *L. reuteri***. (i) Electrocompetent cells in which RecT is expressed are transformed with a recombineering oligonucleotide. Transformation efficiencies of >10^5^cfu/mg DNA are required for high recombineering efficiencies. Wavy black lines with a red dot represent recombineering oligonucleotides with multiple non-complementary bases; the green circle represents a bacterial cell; pJP042 is the sakacin-based expression vector that contains recT; the black double helix represents chromosomal DNA; expressed RecT proteins are denoted in the cell. (ii) An oligonucleotide identical to the lagging strand contains multiple non-complementary bases that avoid the mismatch repair system resulting in increased recombineering efficiency. (iii) Viable cells are recovered on antibiotic-free plates and recombinants are detected by a mismatch amplification mutation assay-PCR (MAMA-PCR). Two oligonucleotides (blue) will yield a 1-kb fragment, whereas a third oligonucleotide (red) will only be extended by the polymerase when the mutations are incorporated in the chromosome yielding a second amplicon of 500 bp. As the recombineering oligonucleotide only targets one strand during DNA replication the colonies will be of mixed genotype. The red dot on the chromosome indicates that the mutations are incorporated. (iv) Single colony purification is performed to separate the wild-type genotype from the mutant genotype. (v) MAMA-PCR is repeated as described in section iii to identify a pure genotype mutant. A 1:1 ratio of wild-type and mutant genotypes is suggestive that during replication a single chromosome is being replicated per cell. (vi) The recombineering plasmid pJP042 can now be cured from the mutant strain by passaging bacteria without antibiotic selection to yield a plasmid-free derivative (vii). (Reprinted by permission from Oxford University Press, ^© ^2012).

**Figure 2:**
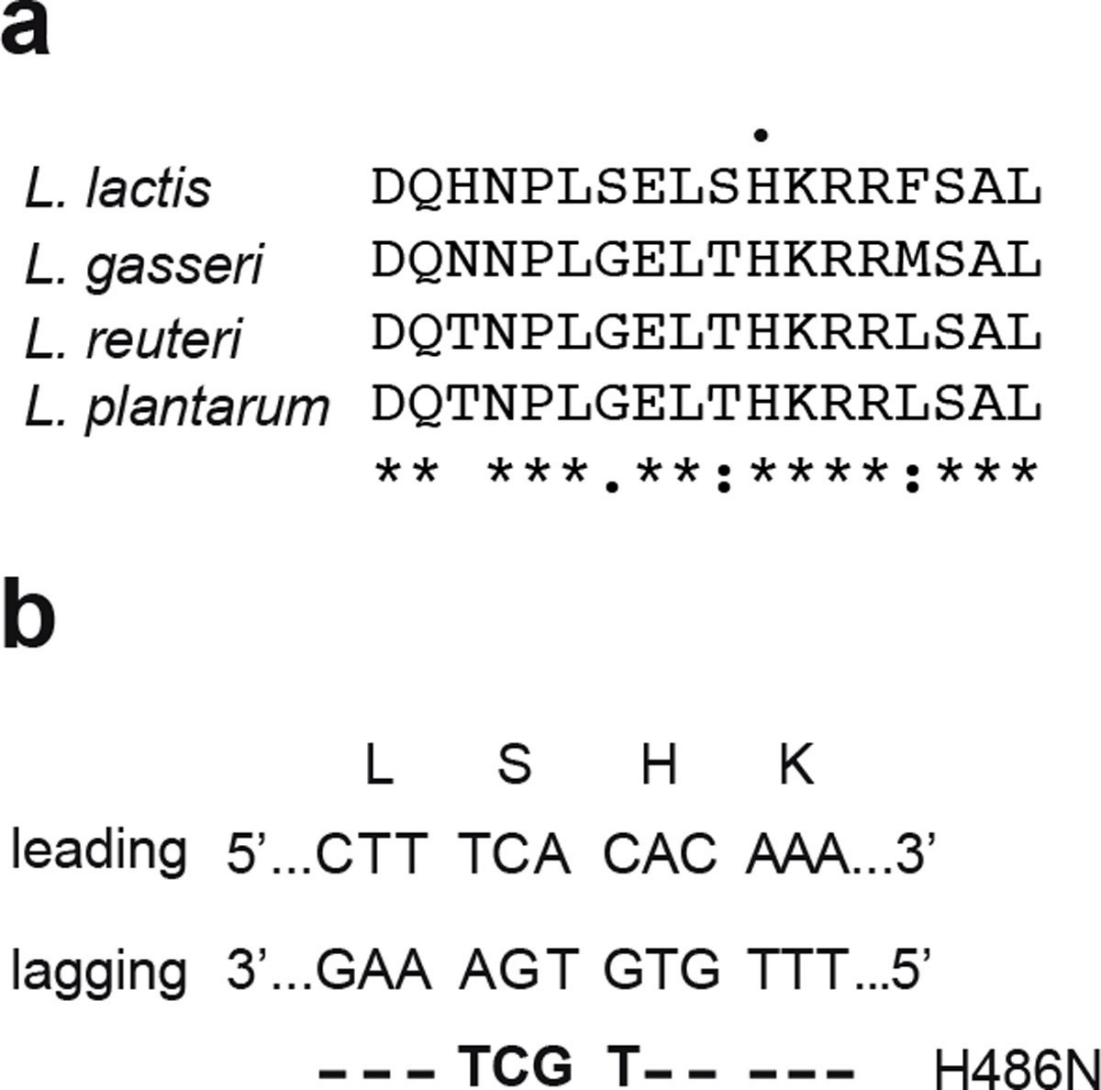
**Base changes in the *rpoB *gene leading to an amino change of a conserved histidine gives rise to rifampicin resistance**. a. Local alignment of RpoB from four LAB strains that highlights a conserved histidine (highlighted by a single black dot above the sequence) which has been found to yield rifampicin resistance in these LAB strains as well as in *Bacillus subtilis *and *E. coli *when another amino acid is substituted in its place. Asterisks (*) below the alignment indicate a single conserved residue, a colon (:) represents residues which share strongly similar properties and a period (.) indicates residues that have weak similar properties. b. An example of how to design an oligonucleotide that will incorporate four consecutive mismatches while only altering the histidine residue. The leading and lagging strands are indicated while the oligonucleotide is shown below in bold. **- **indicates the nucleotide found in the lagging strand is identical to the nucleotide in the oligo and the base mismatches are indicated in bold letters. The successful incorporation of the oligonucleotide into the chromosome will eventually lead to the alteration of the serine codon from TCA to AGC, which still codes for serine while the histidine codon (CAC) is changed to AAC (asparagine).

A graphical overview of SSDR is presented in Figure 1. When establishing SSDR, the most convenient approach is to modify the DNA of a gene that results in a selectable phenotype to easily assess the level of recombinants. We found that mutating the *rpoB *gene and selecting for rifampicin resistance in various LAB strains can efficiently determine the efficacy by which the recombineering oligonucleotide is incorporated in the chromosome. In order to identify mutations that yield a rifampicin-resistant phenotype we analyzed the *rpoB *sequences of several natural rifampicin-resistant colonies, and confirmed that mutations in a codon coding for a conserved histidine residue results in a rifampicin-resistant phenotype (see Figure 1) in all four lactic acid bacteria investigated [19]. Depending on the adjacent codon it may be possible to make additional silent mutations resulting in 4 adjacent mismatches which evades MMR, yet the mutations will result in only a single amino acid change (Figure 2). In *L. reuteri *6475 that was not possible, and we determined experimentally whether additional amino acid changes yielded a viable rifampicin resistant phenotype [19].

To establish SSDR in LAB, a good place to start would be to transform 100µg of an 80mer oligonucleotide that is identical to the lagging strand of replication, with the exception of 4-5 adjacent, centrally located non-homologous bases which are needed to evade MMR and to incorporate the mutations to yield a rifampicin-resistant phenotype. The number of rifampicin-resistant cells relative to the total number of viable cells indicates the percentage of recombinants. This approach allows further optimization of a variety of parameters, including expression of different recombinases, duration of recombinase expression, efforts to reduce degradation of oligonucleotides by the host nucleases (eg. PT linkages), oligonucleotide length and concentration. We direct the readers to recent reports that describe a variety of parameters that can be optimized for SSDR [10,14,16,19,26,27].

We do not recommend targeting a gene that allows screening based on a color phenotype, such as ß-galactosidase when plated with X-gal as a substrate. With SSDR the oligonucleotide directed mutation(s) are incorporated in only one of the strands during DNA replication. In both *L. reuteri *6475 and *L. lactis *NZ9000 we have noted that, when targeting a gene for mutagenesis by SSDR that cannot be selected, single colonies on an agar plate are almost always a mixed population of cells representing both wild type and mutant genotypes. Either during the recovery phase after electroporation the chromosomes have not segregated, or the chromosomes have segregated but due to a clumpy phenotype or chain formation, single colonies represent a mixed cell population. Therefore, a blue-white screen to identify cells in which the ß-galactosidase gene has been inactivated is not a good choice since it will be impossible to quantify the absolute level of SSDR.

#### Methods to screen for mutations when no selection is performed

Once the SSDR efficiency is high enough to allow identification of mutations without the need for selection, genes can be targeted to make multiple mutations to incorporate a stop codon, for example. In both organisms, identification of mutants can be established by MAMA-PCR [28], or PCR followed by a restriction digest if a restriction site is incorporated. For gene inactivation by incorporation of an in-frame stop codon in both *L. lactis *and *L. reuteri*, we design the recombineering oligonucleotide such that in addition to a stop codon additional mutations yield a restriction endonuclease recognition site that is unique compared to the 500 bp flanking regions. To identify a mutant genotype we apply MAMA-PCR that uses three oligonucleotides in a single reaction (Figure 1). We design the flanking oligonucleotides such that these are located 500 bp upstream and downstream of the target site, thus yielding a 1,000 bp amplicon. When the recombineering oligonucleotide is incorporated, four or five bases on the 3'-end of the MAMA oligonucleotide can anneal to the mutated sequence and will form a 500 bp amplicon with one of the flanking oligonucleotides. Once the mutation is generated we isolate a pure mutant genotype by streaking positive colonies for single isolates. In rare cases the MAMA oligonucleotide may not work, and we can repeat the screen with only the flanking oligonucleotides. The 1,000 bp amplicon can subsequently be subjected to restriction digestion analysis to identify colonies that contain the mutant genotype.

SSDR also allows construction of a single base change. A single C·C mismatch may not be recognized by the MMR but, unless a novel restriction site is generated, genotypic detection of a single base change will be challenging. MAMA-PCR will be unreliable as we noted that at least 3-4 non-homologous bases on the 3'-end of the oligonucleotide need to be present in order to obtain reliable and reproducible results. Even with three non-homologous bases we often noted false-positive amplicons, and although PCR settings may be optimized, this is not a desirable approach for high-throughput screenings. Instead, a single base change can be generated in a 2-step SSDR approach. In the first round 5 adjacent bases are mutated, followed by identification of the mutant, and purification of the pure genotype. This mutant can subsequently be subjected to a second round of SSDR in which 4 out of the 5 bases will be reverted to the wild type sequence, and also this can be screened by MAMA-PCR. The end product will be a single base mutation in the chromosome. In both steps the MMR will be evaded, and mutants can be screened in a high-throughput manner by PCR screen.

### Future developments in precision genome engineering in LAB: improving SSDR using CRISPR-Cas

A major limitation in adapting SSDR to other bacterial hosts is the ability to achieve efficiencies that would allow the modification of any site in the genome and easily recover the mutants without selection. Recently, a novel method for eliminating cells that have not incorporated a mutation into the genome using the Cas9 endonuclease, which is associated with a type-II clustered regularly interspaced short palindromic repeats (CRISPR) locus from *Streptococcus pyogenes*, has been described [29-33]. Although a thorough discussion of the entire family of the CRISPR-Cas adaptive immune system in bacteria is beyond the scope of this review, we briefly describe the salient features of type-II CRISPR-Cas systems and how it has been adapted for use in precision genome engineering.

CRISPR-Cas systems function to keep foreign DNA from invading into the cell and they do so by incorporating segments of the foreign DNA (denoted as protospacers) into the genome (eg. phage DNA), likely via the activities of Cas1 and Cas 2 that are universally found associated with CRISPR-Cas systems (for reviews see [34-36]). These newly incorporated sequences, termed spacers, are located between short palindromic repeat sequences in an ordered array. In addition to the spacer sequence, a short 2-5 bp protospacer associated motif (PAM) located adjacent to the spacer is found and is critical for recognition of incoming foreign DNA by the CRISPR-Cas system. Once a CRISPR locus has acquired a spacer sequence that targets a particular phage, type-II CRISPR-Cas systems have a RNA-directed endonuclease (Cas9) that will cleave any DNA within the cell that is identical to the spacer. While all types of CRISPR-Cas systems require the crRNA to direct target cleavage, type II systems require a second RNA denoted trans-activating crRNA (tracrRNA). Although one function of the tracrRNA is to assist in the generation of fully mature crRNA, both RNA molecules are required for Cas9 cleavage of target DNA. The two RNAs interact with Cas9 to direct the endonuclease to DNA sequences with 100% identity to the crRNA and that also have the correct PAM sequence that is recognized by Cas9.

Because CRISPR-Cas systems cannot discriminate between foreign DNA and the host DNA like restriction systems, they can create double strand breaks in the chromosome of the host cell (often termed "autoimmunity") [37]. The ability to home the Cas9 endonuclease to specific targets in the chromosome has been exploited to generate loss of function mutations in eukaryotic systems. Once Cas9 introduces a double-stranded break in the DNA, eukaryotic organisms do not always repair the damage back to the wild-type sequence due to pathways such as non-homologous end joining - NHEJ. NHEJ can join non-homologous ends that can result in insertions or deletions at the target site, which most often leads to loss of function mutations.

**Figure 3:**
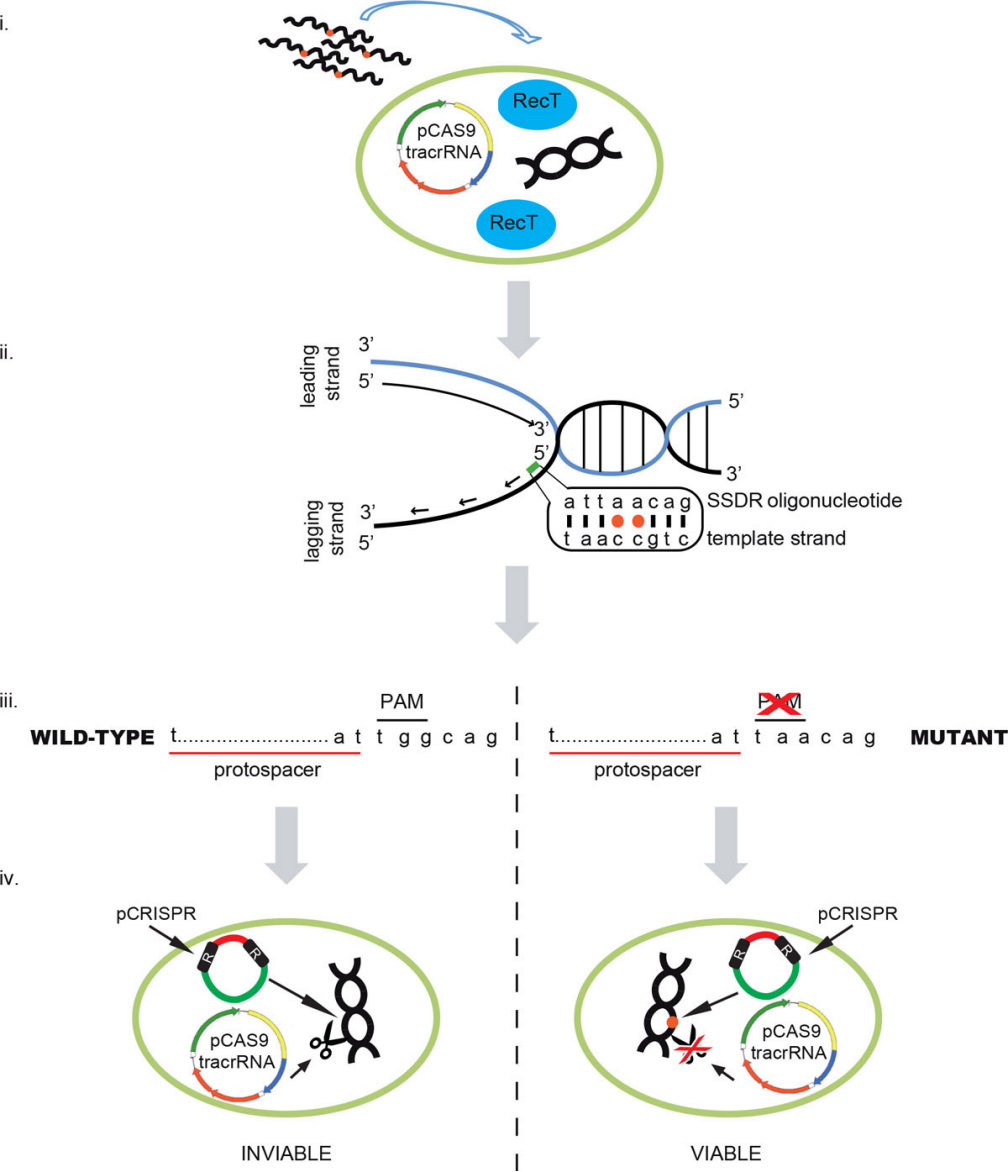
**SSDR/Cas9 genome engineering**. (i) A bacterial strain is transformed with a plasmid containing a Cas9 endonuclease along with a tracrRNA. RecT activity can be provided either in trans on another plasmid or if the bacterium has a RecT protein in the native chromosome endogenous recombinase activity may be sufficient if recombineering activity is high enough. The strain is made electrocompetent and an oligonucleotide containing the desired mutation to be incorporated (denoted by red circle). (ii) Incorporation of the oligonucleotide (green) ultimately results in the alteration of G-G for T-T, which will disrupt the PAM site (iii). Note that the PAM in this example (NGG) is variable depending on the source of Cas9 utilized in the experiment. (iv) Electroporation of a plasmid containing a crRNA targeting the chromosome into the population of recombineered cells. The crRNA will direct Cas9 to cleave the chromosome of the wild-type cells, while mutant chromosomes will not be cut. By selecting for cells that contain the crRNA plasmid by antibiotic selection, only cells that have acquired the mutation via recombineering and avoid Cas9 cleavage will survive.

More than 120 reports of targeting CRISPR to generate loss-of-function mutations in eukaryotic systems were published last year. However, bacteria faithfully repair double strand breaks (or in some cases have poor NHEJ activities) and thus simply targeting CRISPR-Cas9 to a specific locus is not sufficient to generate mutations. Marraffini and co-workers combined SSDR technology in *E. coli *with CRISPR-Cas9 to generate mutations in the chromosome that conferred streptomycin resistance (*rpsL*). They found that ~65% of the cells that survived dual SSDR/Cas9 modification incorporated the mutation into the correct location of *rpsL*, a 1,000-fold improvement of SSDR (the mutation they constructed was subject to MMR and thus had reduced SSDR efficiency). The efficiency of CRISPR-Cas9 in this experiment is not high enough to allow isolation of recombinants in which SSDR is not efficient enough to generate mutations without selection. However, it is likely only a technical hurdle to improve CRISPR-Cas9 in bacteria such that >99.99% of non-recombinants can be eliminated. Once this is achieved, almost any organism that low SSDR activity (either naturally or by inducing the expression of RecT) will be able to be modified at nearly any site in the chromosome. A schematic for how SSDR coupled with CRISPR-Cas9 would function in precision genome engineering is shown in Figure 3.

#### Future prospects and directions

SSDR offers the ability for precision genome engineering without the need for antibiotic selection. While several challenges remain, the promise of using SSDR in combination with CRISPR-Cas9 to eliminate non-recombinants means that genetic modification without the need for antibiotic selection of many different bacterial strains and species is likely to be available in the near future. The identification of novel Cas9 proteins with different PAMs will expand the number of sites available in a genome for engineering [38,39]. Bacteria that harbor *recT *homologs in their chromosome, like *L. reuteri *6475, likely will not require the introduction of RecT on a plasmid when combined with Cas9 counter-selection due to the high level of inherent SSDR activity in this strain. The development of inducible expression systems will be required for bacterial strains in which existing technology does not work and have no endogenous SSDR activity.

Currently there are three bacterial species in which SSDR can be performed to identify mutations without the need for selection (Mycobacterial sp. are the fourth but require co-selection of cells that have taken up DNA to find recombinants). A major challenge moving forward with SSDR in LAB will be the successful establishment of this technology in industrially and medically important strains of LAB. Interestingly, *E. coli *and *L. lactis *NZ9000 are lab-adapted strains that have been passaged in the laboratory for decades and have similar optimization strategies in SSDR. The lagging strand bias, the ability of PT linkages to increase, and the overall efficiency of SSDR similar between *L. lactis *and *E. coli*. However, the incorporation of PT linkages does not improve SSDR efficiency in *L. reuteri *and in fact may inhibit SSDR, even though SSDR efficiency is quite a bit lower than in the lab adapted strains [16]. *L. reuteri *6475 is a human isolate and has had little exposure to laboratory conditions. Working with other closely related *L. reuteri *strains we find a wide range of recombineering activity, indicating that other, yet unknown, parameters for optimizing SSDR remain to be identified. For example, the induction of temperate phages by the introduction of large amounts of oligonucleotide may be a major hurdle to overcome when working with strains that have not been passaged in the laboratory.

SSDR now provides us with the ability to make genome changes, yielding an in-frame stop codon or a single amino acid change, leaving no trace of foreign DNA in the cell. In fact, using SSDR we can mutate a single base whereas strains that have undergone a long selection procedure will most likely contain multiple base changes. Taken this into consideration, a GMO strain engineered by SSDR should be subjected to at least the same risk assessment procedures as a non-GMO derivative which may contain multiple bases changes for which we do not know how they change gene function(s) and phenotypes.

Although GMO regulations differ in different regions of the world, the ability to construct strains that are genetically identical to strains that were "selected naturally" or modified using non-GMO technologies demonstrates that simply using a plasmid to construct a strain does not yield a strain that is inherently any more dangerous than non-GMO strains. Consider the following hypothetical example: a researcher would like to generate a probiotic bacterium that is resistant to high concentrations of human bile. To isolate such a resistant mutant, the cells are plated in the presence of bile, a bile resistant mutant is isolated and its genome is sequenced. A single base pair alteration in the hypothetical gene *bilE *is identified as the mutation yielding the bile resistant phenotype. Utilizing SSDR technology, this identical mutation in *bilE *is constructed in the probiotic organism with the subsequent plasmid containing *recT *removed. Deep sequencing technology allows us to confirm that no foreign DNA remains in the cell, and no additional mutations have occurred. Thus we have two strains that, by any measure, are genetically 100% identical. If the reason for designating a strain as GMO is for public safety, then there were no reason to believe that in the above example a strain mutated by SSDR would be less safe than a genetic identical non-GMO derivative.

In summary, SSDR provides researchers with a valuable tool with which to probe functions of industrially and medically important strains of LAB. The revolutionary aspect of SSDR is the fact that virtually any region of the genome can be altered, which means that the rate-limiting step in LAB genetics will soon be figuring out what types of mutations you want to construct rather than the genetic tools available for study. Combined with other emerging technologies, such as CRISPR-Cas9, we envision a future in which strains can be engineered in ways that are not only beneficial but also more safely constructed by avoiding unwanted mutations associated with "non-GMO" approaches that allow the use of mutagens in the selection of bacteria with desirable phenotypes.

## Competing interests

The authors declare that they have no competing interests.

## Supplementary Material

Additional file 1 a) Identification of the lagging strands of replication in a bacterial genome, and SSDR oligonucleotide design. b) (i) A systematic approach to design a SSDR oligonucleotide to incorporate an in-frame stop codon. Click here for file

## Notes

This article has been published as part of *Microbial Cell Factories *Volume 13 Supplement 1, 2014: Proceedings of the 11th International Symposium on Lactic Acid Bacteria. The full contents of the supplement are available online at http://www.microbialcellfactories.com/supplements/13/S1. Publication charges for this supplement were funded by the Stichting Symposium on Lactic Acid Bacteria. The articles have undergone the journal's standard peer review process for supplements. EJ is employed by Chr Hansen A/S, is a shareholder in Chr Hansen A/S; and has applied for patents relating to the content of an article which he did not edit but which forms part of this supplement. MK is an employee of Nizo. The other Supplement Editors declare that they have no competing interests.
