# Supplementary material for: Precision genome engineering in lactic acid bacteria
Source: Microb Cell Fact. 2014 Aug 29;13(Suppl 1):S10. doi: 10.1186/1475-2859-13-S1-S10 (PMC4155826; doi:10.1186/1475-2859-13-S1-S10)
Supplement: Additional file 1 — a) Identification of the lagging strands of replication in a bacterial genome, and SSDR oligonucleotide design. b) (i) A systematic approach to design a SSDR oligonucleotide to incorporate an in-frame stop codon. [file 1475-2859-13-S1-S10-S1.docx]

**Additional file 1**

**
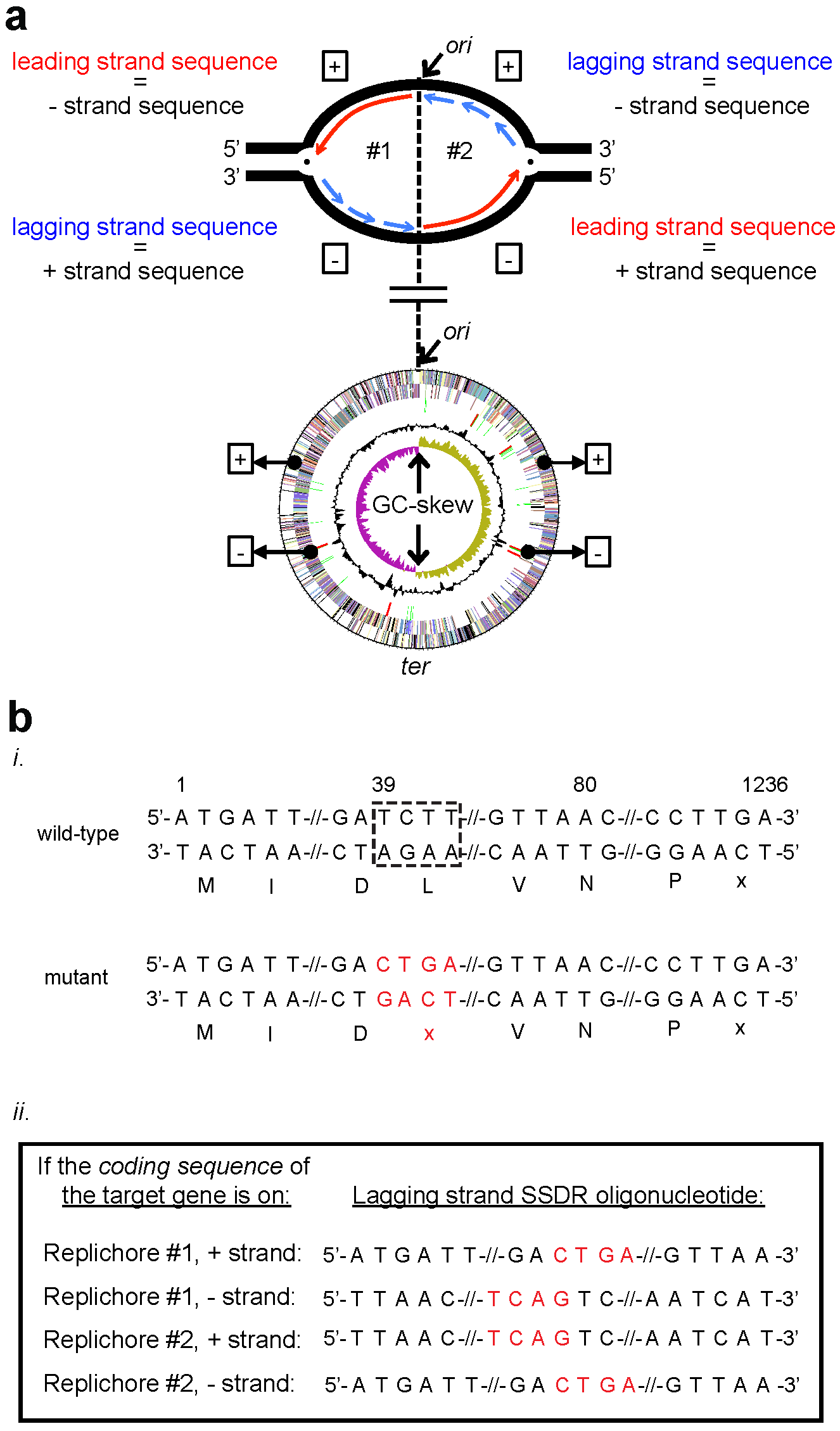
**

**a)** **Identification of the lagging strands of replication in a bacterial genome, and SSDR oligonucleotide design**. Top panel: Replication forks fired at the origin of replication (*ori*) region are depicted in the top panel. The black lines represent the template DNA with the proximal ends indicated by 5’ and 3’. + and - represent the positive and negative DNA strands, respectively. DNA replication is bi-directional, resulting in two replichores (#1 and #2) separated in the figure by a dashed line. Each replichore has its own replication fork (•) where the DNA strands are unwound and separated to expose single-stranded template DNA. In replichore#1, when the positive strand serves as the template for replication the newly synthesized strand (identical to the negative strand) represents the leading strand of replication (red arrow). When the negative strand in replichore#1 serves as template, replication is discontinuously because the DNA polymerase moves away from the replication fork. As the DNA strands at the replication fork are unwound and separated, a new primer for replication needs to be synthesized which is extended by the DNA polymerase into a new strand until it reaches the primer located downstream, and this process if repeated during replication. These short DNA fragments synthesized are called Okazaki-fragments (blue arrows) and represent the lagging strand of replication. The lagging strand sequence in replichore#1 is identical to the positive template strand of replichore#1. In replichore#2 the leading and lagging strands are swapped relative to replichore#1 due to the polarity of DNA.

Bottom panel: Additional factors that can be used to identify the lagging strand of replication. This genome representation (*L. reuteri* JCM1112) has five rings: (from outside to center) genes coded on the positive strand, genes coded from the negative strand, RNA genes, GC-content, and the GC-skew. In most bacterial chromosomes a majority of genes are oriented in the same direction as the leading strand replication to avoid conflicts between replication and transcription, thus one can easily deduce that the strand with fewer genes is identical to the lagging strand. Looking at replichore#1 (left half of chromosome) the positive strand has fewer genes present compared to the negative strand, and thus one can predict that the positive strand sequence is identical to the lagging strand. Thus gene density is often an indicator leading and lagging strand designation. Another predictor that separates the two replichores and identifies where the leading and lagging strands transition is the GC-skew. The leading strand has an abundance of G over C compared to the lagging strand and is reflected by a positive and negative value in the GC-skew, respectively (see inner ring). This is important to note as the *ori* and the *ter* are not located in all bacterial chromosomes at 12 o’clock and 6 o’clock, respectively, and the GC-skew can be useful to predict both *ori* and *ter.*

**b)** **(i) A systematic approach to design a SSDR oligonucleotide to incorporate an in-frame stop codon.** First, a sequence file is prepared containing the coding DNA sequence of the target gene. In the example listed (top, wild-type), double-stranded DNA sequence of specific regions from a 1236 base pair fictive gene are shown. Above the sequence are positions of the relevant bases shown, indicated with numbers where base 1 represents the first base of the first codon of the fictive gene. The dashed boxed region starting at base 39 represents the region to be mutated, and base 80 and 1236 represent the proximal end of the SSDR oligonucleotide and the coding sequence of the fictive gene, respectively. Directionality of the sequence is indicated with 5’ and 3’. Below the sequence, central to each codon, is the corresponding amino acid shown. Stop codon is indicated with x. Sequences not shown are indicated with the symbol -//-. For optimum SSDR efficiency, an oligonucleotide should be designed that evades the hosts mismatch repair system. In both *L. reuteri* and *L. lactis* this can be achieved by generating four adjacent mismatches. In this example, we will replace the wobble base of the triplet GAT (wobble base of this codon is base 39 as indicated in the dashed box), and three bases of a triplet coding for leucine (dashed box, CTT) with the bases CTGA (bottom, mutant sequence, highlighted in red) that will result in a silent mutation and an in-frame stop codon. We have successfully inactivated multiple genes in *L. reuteri* and *L. lactis* via this methodology. When designing these experiments, we find it most convenient if we generate the sequence files *in-silico*. Note that care should be taken that the anticipated mutations do not result, combined with the flanking sequence, in a sequence that resembles a ribosomal binding site, as this may result in translation of a truncated protein if an alternative start codon is located downstream. (ii) there is strong lagging-strand bias for optimal SSDR oligonucleotide incorporation (see text for more details) and therefore the SSDR oligonucleotide needs to be identical, with exception of the mutations to be incorporated, to the lagging strand of replication. To identify the lagging strand sequence of the target gene, we first must identify on which replichore the gene is located, and from which strand on the chromosome the gene is coded. Searching a closed genome of the organism to be mutated with the basic local alignment search tool (BLAST) using your target gene sequence as a query will provide you with this information. If no closed genome is available for your organism of interest, the contig sequence of the target gene may give insight whether the target gene is coded from the leading or lagging strand of DNA replication because the majority of the genes are coded from the leading strand (see legend Supplementary Figure 1a, bottom panel, for more details). The box lists the SSDR oligonucleotide sequences identical to the lagging strand when target gene is located on each of the replichores and strands. If the target gene is located on replichore #1 (+ strand) or on replichore #2 (- strand), the coding sequence of the target gene is identical to the lagging strand. Alternatively, if the target gene is located on replichore #1 (- strand) or on replichore #2 (+ strand) the non-coding sequence is identical to the lagging strand. The mutant sequence generated *in-silico* (see i) can now be used to obtain the SSDR oligonucleotide sequence. We recommend to copy 80 bases of the coding sequence of the mutant, and reverse complement if needed, to obtain the SSDR oligonucleotide sequence. We routinely order SSDR oligonucleotides at 100nmol scale (lyophilized, IDT-DNA), and dissolve the oligonucleotide in sterile milli-Q water to a final concentration of 20µg/µl.
